# Management of patients with giant basal cell carcinoma during SARS COV2 outbreak in Italy

**DOI:** 10.1002/tbio.202200009

**Published:** 2022-07-26

**Authors:** Carmen Cantisani, Raimondo Rossi, Steven Paul Nisticò, Martina Vitiello, Francesca Farnetani, Luigi Bennaro, Giovanni Pellacani

**Affiliations:** ^1^ Department of Dermatology and Venereology, UOC of Dermatology, Policlinico Umberto I Hospital Sapienza Medical School of Rome Rome Italy; ^2^ Department of Health Sciences Magna Grecia University Catanzaro Italy; ^3^ Dermatology Department University of Modena and Reggio Emilia Modena Italy

**Keywords:** basal cell carcinoma, giant basal cell carcinoma, imiquimod, immunomodulator agent, superficial basal cell carcinoma, topical therapy

## Abstract

Basal cell carcinoma (BCC) is the most frequently occurring type of all cancers, and represents 80% of all skin cancer. The estimated lifetime risk for BCC in the white population is between 33% and 39% for men and 23% and 28% for women. Its incidence doubles every 25 years and is increasing in the young population. Death is uncommon and seems to decrease in the last years, probably due to early and better diagnosis. BCC arises from abnormal and uncontrolled growth of basal cells. It is a slow‐growing tumor, therefore usually curable at an early stage with surgery or alternative treatment, such as cryotherapy, laser, photodynamic therapy, retinoids and topical agent like 5‐Fluorouracil cream, imiquimod cream, and so forth. Topical treatment of superficial basocellular carcinoma is a viable option, when surgery is not an advisable treatment, especially in the case of giant basocellular carcinoma. In this subtype, imiquimod 5% cream can be a safe and effective treatment, but there are few reports in available literature. We present our case series of eight patients with superficial giant basocellular carcinoma successfully treated with imiquimod 5% cream, which showed clinical improvement after 8 weeks of treatment.
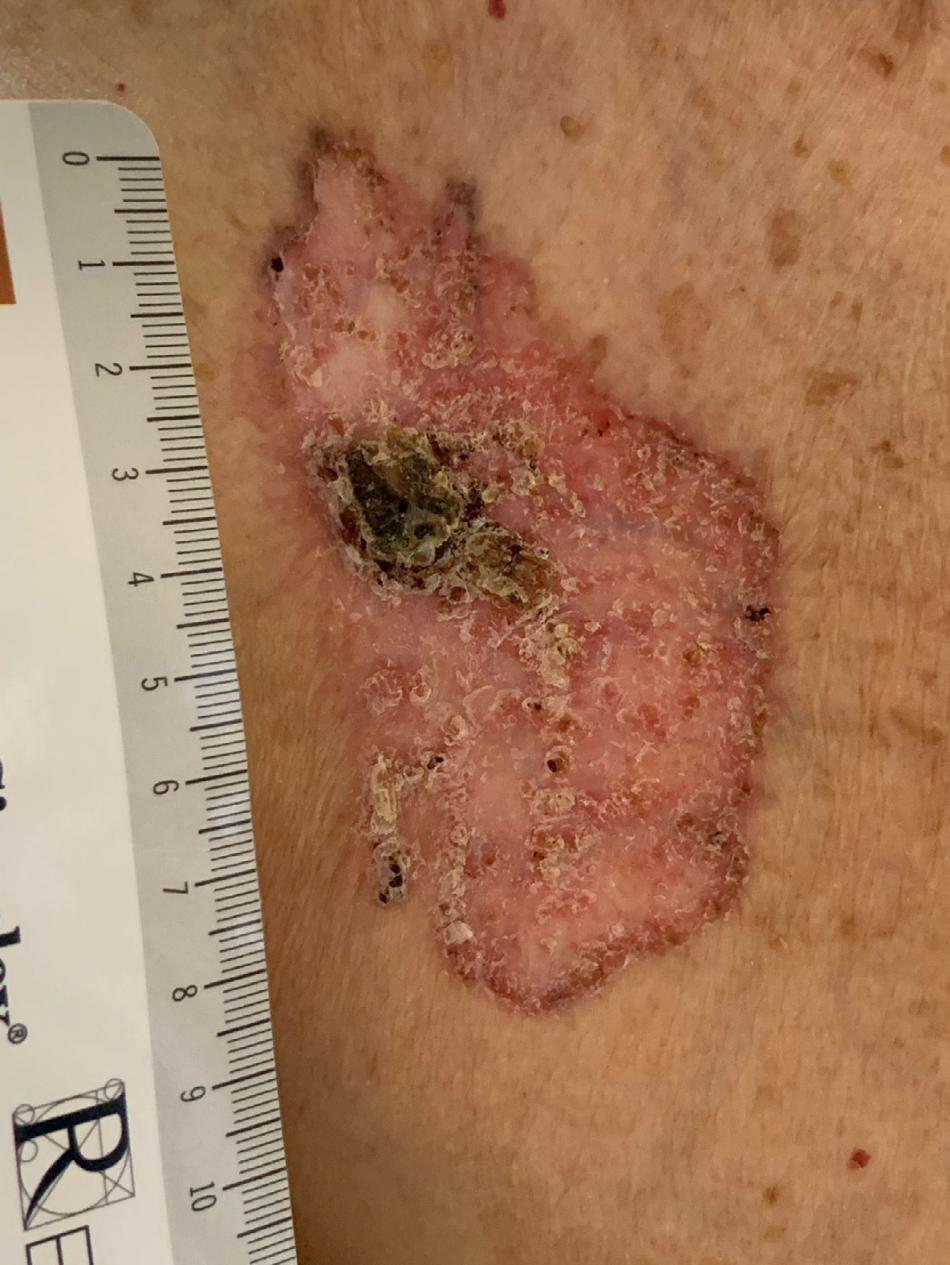

## Graphical abstract

Basal cell carcinoma (BCC) is the most frequently occurring type of all cancers, and represents 80% of all skin cancer. The estimated lifetime risk for BCC in the white population is between 33% and39% for men and 23% and 28% for women. BCC is a slow‐growing tumor, therefore usually curable at an early stage with surgery or alternative treatment, such as cryotherapy, laser, photodynamic therapy, retinoids and topical agents. Topical treatment of superficial basocellular carcinoma is a viable option, when surgery is not an advisable treatment, especially in the case of giant basocellular carcinoma. In this subtype, imiquimod 5% cream can be a safe and effective treatment, but there are few reports in available literature. We present our case series of eight patients with superficial giant basocellular carcinoma successfully treated with imiquimod 5% cream, which showed clinical improvement after 8 weeks of treatment.

## 1 INTRODUCTION

Basal cell carcinoma (BCC) represents the most common malignant tumor; in particular, it is one of the most common malignancies of the skin [1, 2, 3]. It arises from a mutation resulting in an activation of the Hedgehog (HH) pathway, and several subsequent oncogenic hits will be needed for the development of an invasive form. It has been postulated that tumor cells are reprogrammed and dedifferentiate into a precursor cell resembling an embryogenic hair follicle progenitor [3]. Major risk factors for the development of BCC are ultraviolet light exposure and genetic predisposition [2]. Nevertheless, specific etiological factors have not been discovered yet. The diagnosis of BCC poses a relevant concern for the patients because of its malignant potential. In most cases, surgical excision and regular follow‐up are satisfactory to eradicate a single lesion, mainly due to its low hematic and lymphatic metastatic rate and slow growth rate [2].

The histopathological classification of BCC has important prognostic and therapeutic implications. According to the recent European position paper, correlation with clinical characteristics is necessary to determine its risk and therapeutic approach [4]. BCC's that exceed 5 cm in diameter are defined as giant BCC (GBCC) by the American Joint Committee on Cancer [1, 3, 4]. GBCC's are infrequent and represent 0.5% to 1% of all BCCs, and have a higher rate of metastatic disease and local invasion [1, 4]. The main factors that influence their size, at time of presentation, seem to be patient neglect with consequently late diagnosis, and increased biologically aggressive behavior, the cause of which is yet to be determined [1, 2, 3, 4, 5, 6, 7, 8, 9]. We describe a case series of elderly patients with superficial GBCC successfully treated with imiquimod 5% cream.

## 2 CASE SERIES

**FIGURE 1:**
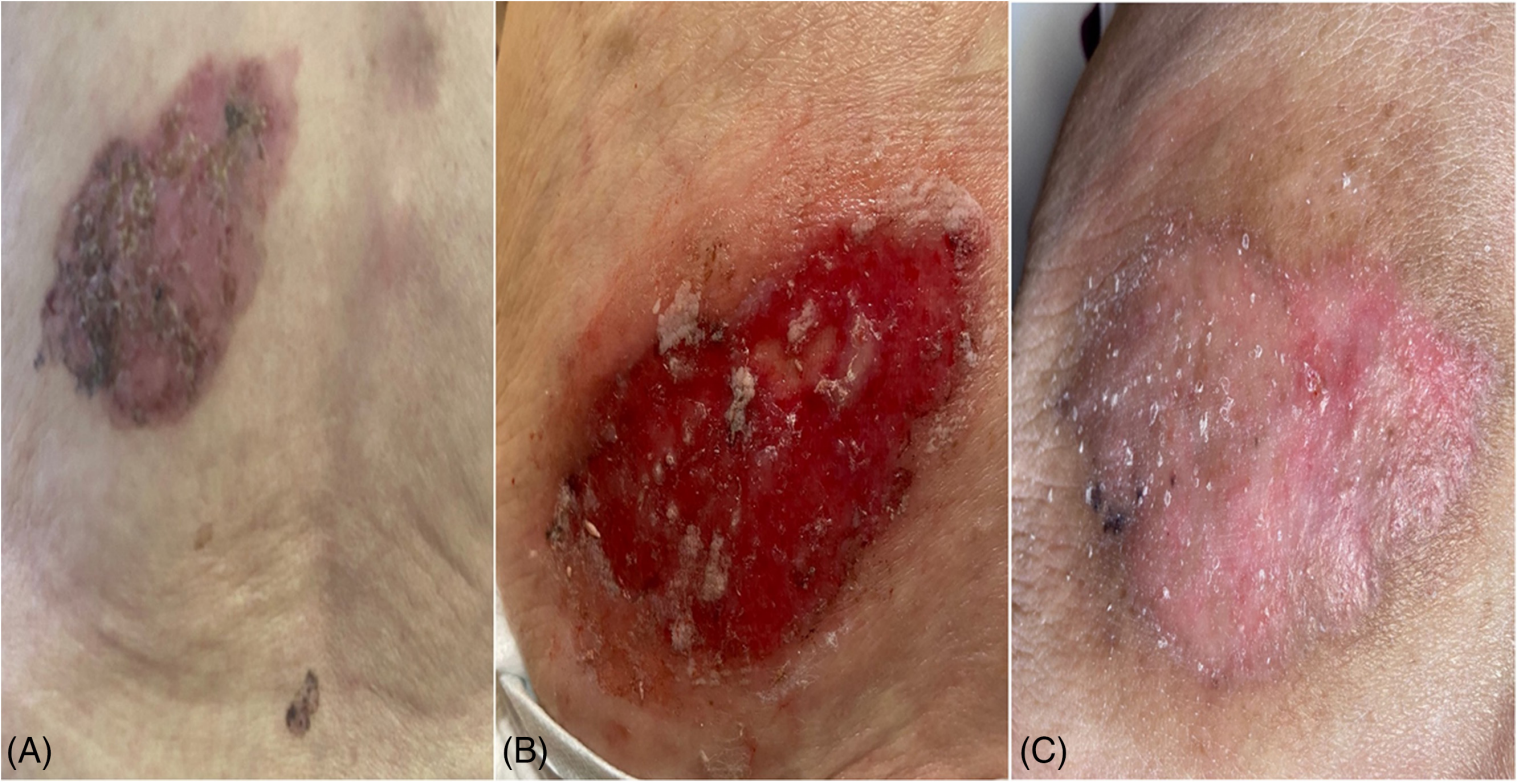
Superficial giant BCC on the left posterior trunk of a 96 years old female patient. A, Before treatment; B, Severe local skin reaction during treatment at 4 weeks; C, After 8 weeks of treatment

**FIGURE 2:**
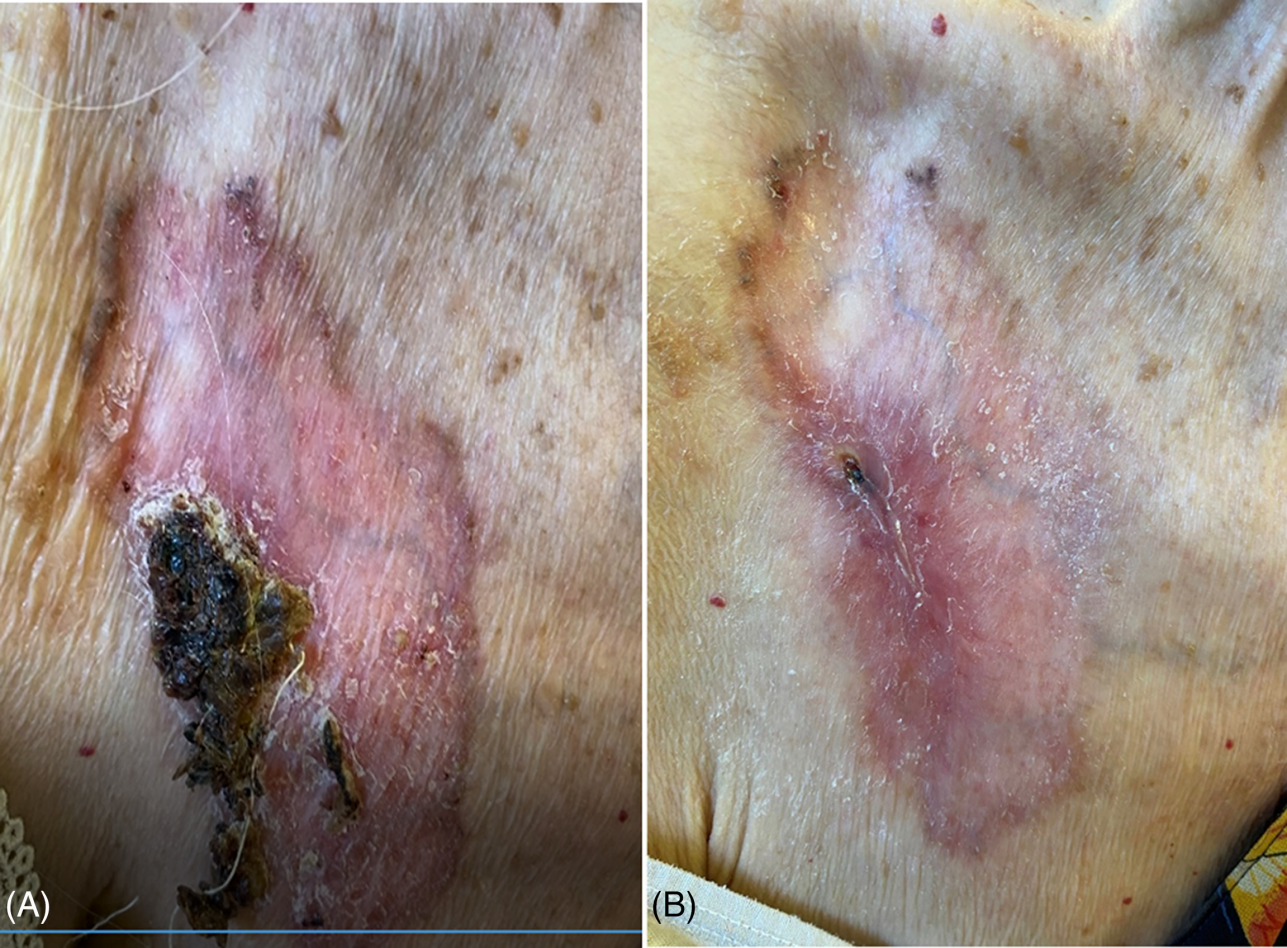
Superficial giant BCC on the right anterior chest of a 90 years old patient. A, Before treatment; B, After 8 weeks of treatment

**FIGURE 3:**
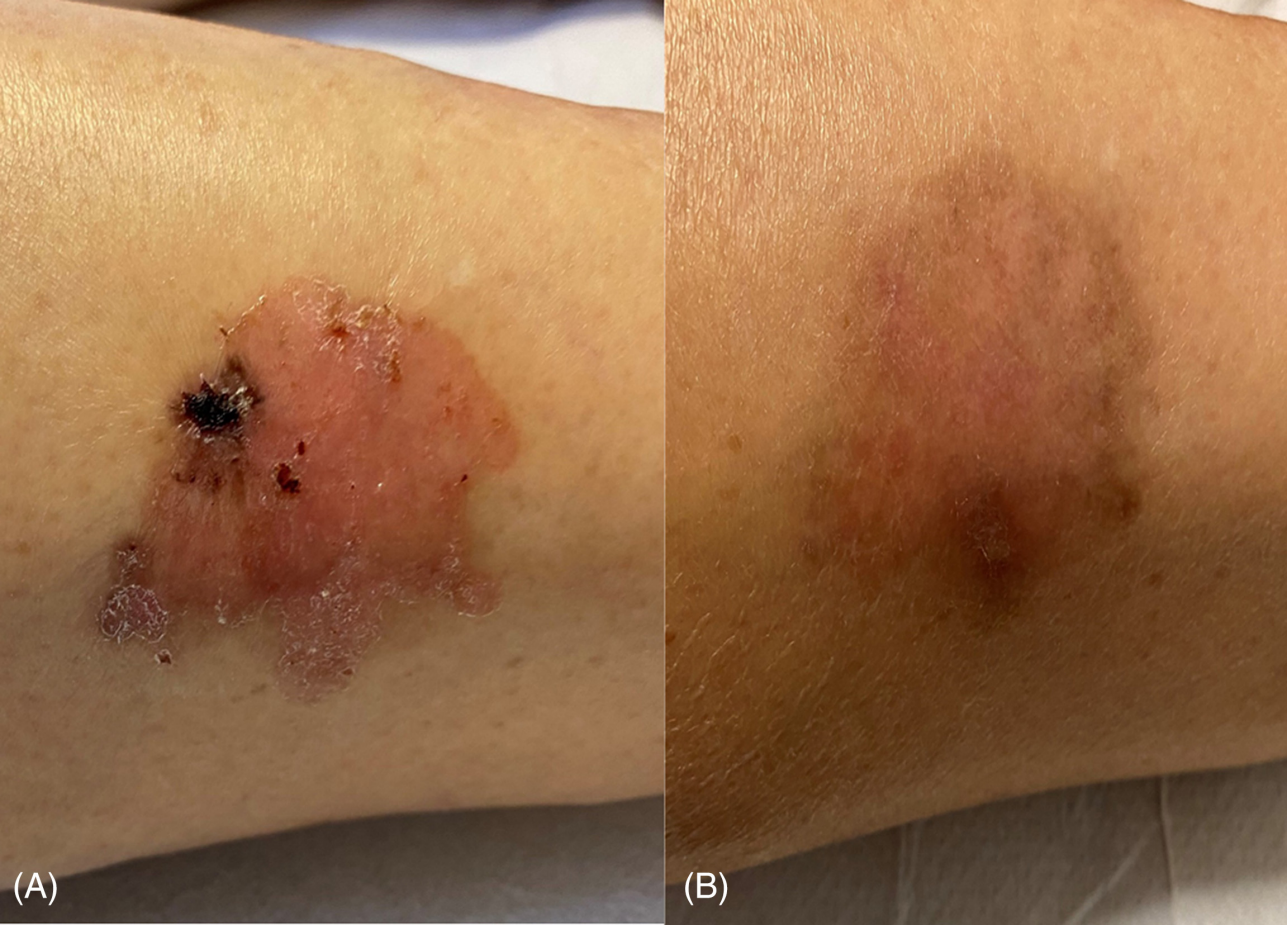
Superficial giant BCC on the right leg of a 78 years‐old male patient. A, Before treatment. B, After treatment

**FIGURE 4:**
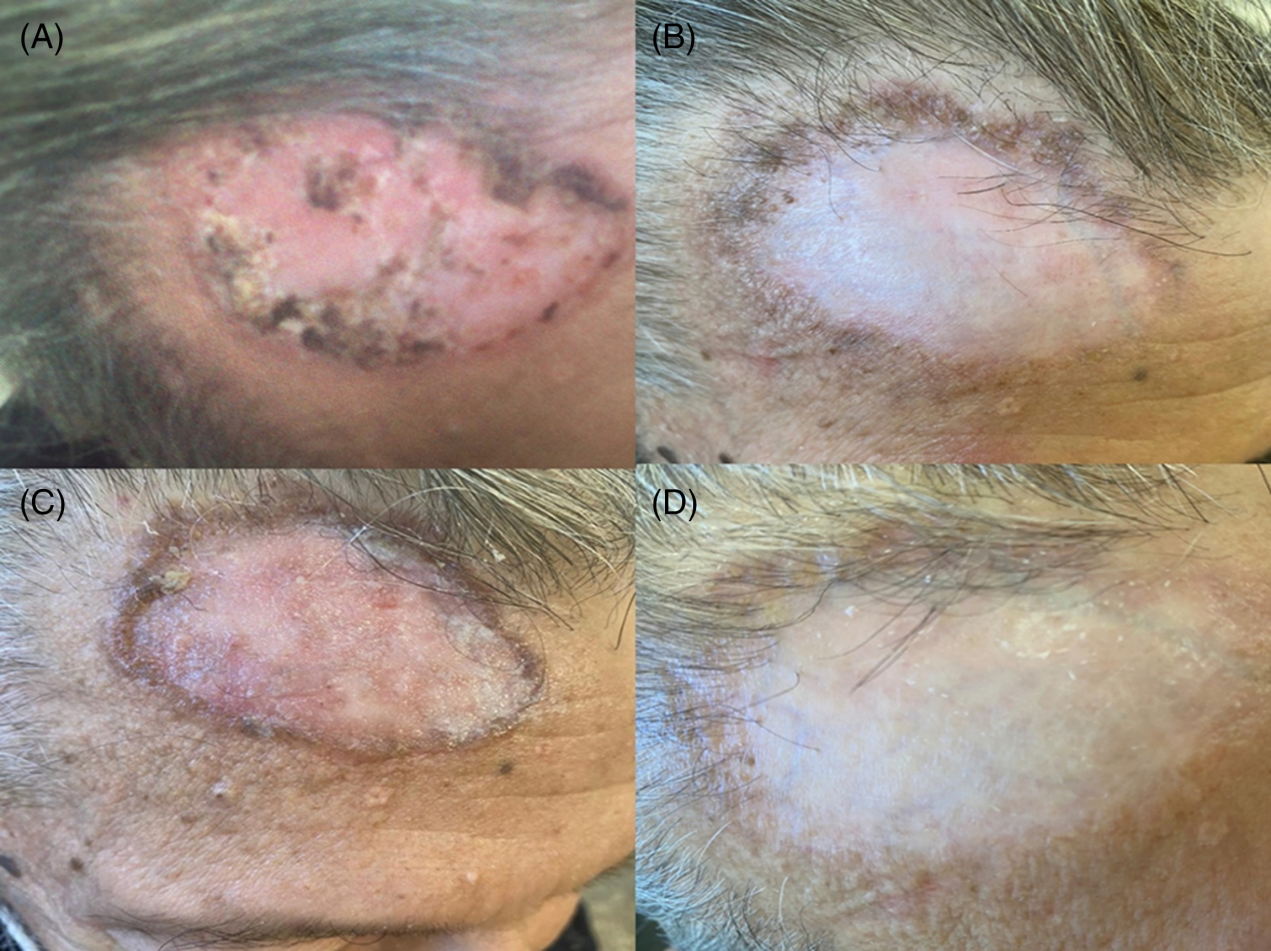
A, T0 72 years old patient with giant Basal Cell Carcinoma (GBCC) of the right frontal region; B, T1 after four sessions of c‐PDT; C, T2 Clinical outcome after 4 weeks of treatment with imiquimod 5% cream; D, T3 Complete resolution after 8 weeks

Eight patients (four female and four male) mean age 84 years old (72‐96), presented with superficial GBCC to our outpatient clinic, after the second wave of the COVID‐19 pandemic. All patients had Fitzpatrick phototype III and other associated small lesions on the face and trunk were also observed. Total brain and body CT‐scan, performed for other indications, showed no distant metastasis. Taking into account their age, lesion diameter and comorbidities, we decided to start topical treatment with imiquimod 5% cream after consulting with caregivers and plastic surgeons. Imiquimod was applied once a day before sleeping, for five consecutive days a week for more than 4 weeks of treatment. Caregivers were instructed that if local skin reactions appeared they could stop application and apply skin relieving medications (boric acid and zinc oxide) and after complete healing resume treatment until complete resolution 8 weeks later (Figures 1, 2, 3). In one patient with giant BCC on the forehead we tried to treat with conventional photodynamic therapy (cPDT) first (4 PDT session, every 30 days), with partial resolution, followed by topical imiquimod 5% cream (Figure 4). Dermoscopic evaluation was performed at baseline and at the end of treatment, no biopsy was made. Clinical improvement was achieved after 8 weeks of treatment, and no recurrences were seen after 6 months in all patients.

## 3 DISCUSSION

Basal cell carcinoma is the most common skin cancer in the elderly white population, mostly due to sun exposure [2, 6]. Recently, its incidence is increasing in young patients even in the absence of genetic disorders. Its incidence has seen a steady rise in the last decades, mostly because of the increased longevity of the general population and actinic damage following chronic sun exposure behavior [2, 6]. Main risk factors are light‐skinned phenotypes, intermittent sun exposure, immunosuppression, family history of BCC or other skin cancer [2, 6]. The main causative factor is ultraviolet (UV) radiation and the gene most often mutated in sporadic BCCs is PTCH1, which encodes the protein Patched 1, an oncosuppressor of essential importance in the Hedgehog pathway [2]. The same PTCH1 gene is mutated at the germline level in the Basal Cell Nevus Syndrome (formerly Gorlin‐Goltz Syndrome) [7]. Its histogenesis is still a matter of debate, although it seems that most BCCs arise from the stem cells located in the hair follicle [2, 8, 9, 10, 11]. This observation could explain why BCC growth is restricted to skin with pilosebaceous units. Clinically, BCC can commonly present as four subtypes: superficial, nodular, morpheaform and ulcerated [2]. The main therapeutic approach is complete surgical excision, and Mohs' micrographic surgery is suggested ‐ although not widely performed ‐ in selected cases in which maximal preservation of tissue and high risk for positive margins are a concern [2]. If left untreated, a BCC can grow and become locally invasive (as in the case of ulcus rodens) and difficult to excise. Residual tumor persistence leading to high recurrence rates is observed especially in high‐risk patients and high‐risk anatomical sites. Therefore, alternative treatment (topical and systemic) can be considered in selected cases [2], such as GBCCs and elderly patients with a short life expectancy and/or with major comorbidities. The burden of the COVID‐19 pandemic on the healthcare system resulted in significant delay in all routine medical performances, including surgical procedures, resulting in a serious lag in the treatment of some patients. This lag, combined with commonly observed patient neglect, can be one of the reasons for an increase in BCC diameter at the time of first dermatological consult. In these cases, topical medication and photodynamic therapy can be valid and effective alternative treatments [12, 13, 14, 15].

In particular, topical therapy with 5% imiquimod cream has been shown to be an effective approach especially for small (<2 cm) superficial BCCs in immunocompetent patients [1, 2, 9], for which it is approved in Europe and the United States. There is limited evidence regarding the efficacy of this regimen for the treatment of nodular BCCs [9]; the reason behind the alternating results is probably that the efficacy of topical therapy depends upon the grade of tumor infiltration and patient compliance.

In the case of GBCCs, available literature offers evidence of successful treatment and long‐term clearance of lesions bigger than 5 cm with topical 5% imiquimod [1], as shown also in our case series.

Imiquimod works by modifying the immune response, enhancing both innate and adaptive immune responses. Specifically, it binds to Toll‐like receptor 7 (TLR7) on anti‐gen‐presenting cells, activating the signaling cascade that stimulates the production of nuclear factor κB (NFκB), promoting APC maturation and enhancing Th1‐type response [9, 10]. It was a valid therapeutic alternative in our patients and local skin reactions were well accepted by patients and caregivers. Following the declaration of the COVID‐19 pandemic by the WHO on 11 March 2020, elective dermatology consultations were limited to urgent cases, but adequately taken photos could be sent via telemedicine, by caregivers, to the dermatologist during home therapy, increasing compliance and therefore efficacy of the suggested treatment. In this pandemic period especially, this regimen allowed patients to be safely treated at home, avoiding hospitalization and monitoring local skin reaction should be done via telemedicine [16]. Allowing patients to access to the hospital at the beginning and after complete healing, reducing the number of visits and the risk for elderly people to become infected.

In our opinion, according with sporadic cases described in literature [17], this treatment needs to be considered as a valid alternative in elderly people with giant BCC, but strict follow up is mandatory.

## FUNDING INFORMATION

This research received no external funding.

## CONFLICT OF INTEREST

The authors declare no conflict of interest.

## Data Availability

none.

## References

[1] K. Peris , M. C. Fargnoli , C. Garbe , R. Kaufmann , L. Bastholt , N. B. Seguin , V. Bataille , V. D. Marmol , R. Dummer , C. A. Harwood , A. Hauschild , C. Höller , M. Haedersdal , J. Malvehy , M. R. Middleton , C. A. Morton , E. Nagore , A. J. Stratigos , R. M. Szeimies , L. Tagliaferri , M. Trakatelli , I. Zalaudek , A. Eggermont , J. J. Grob , European Dermatology Forum (EDF), the European Association of Dermato‐Oncology (EADO) and the European Organization for Research and Treatment of Cancer (EORTC) , Eur. J. Cancer 2019, 118, 10. 10.1016/j.ejca.2019.06.003. 31288208

[2] D. P. Kim , K. J. B. Kus , E. Ruiz , Hematol. Oncol. Clin. North Am. 2019, 33(1), 13. 10.1016/j.hoc.2018.09.004. 30497670

[3] K. Kretzschmar , C. Weber , R. R. Driskell , E. Calonje , F. M. Watt , Cell Rep. 2016, 14(2), 269. 10.1016/j.celrep.2015.12.041. 26771241 PMC4713864

[4] M. T. Fernández‐Figueras , J. Malvehi , P. Tschandl , A. Rutten , F. Rongioletti , L. Requena , H. Kittler , K. Kerl , D. Kazakov , B. Cribier , E. Calonje , J. André , W. Kempf , Study Group Collaborators (Validation Group) , J. Eur. Acad. Dermatol. Venereol. 2022, 36(3), 351. 10.1111/jdv.17849. 34931722

[5] J. C. Purnell , M. G. Jerad , J. A. Brown , S. C. Shalin , Ind. J. Dermatol. 2018, 63(2), 147. 10.4103/ijd.IJD_165_17. PMC5903045 29692457

[6] D. Oudit , H. Oham , T. Grecu , et al., J. Plast. Reconstr. Aesthet. Surg. 2020, 73(1), 53. 10.1016/j.bjps.2019.06.029. 31519500

[7] M. R. Vaca‐Aguilera , E. Guevara‐Gutiérrez , J. G. Barrientos‐García , A. Tlacuilo‐Parra , Int. J. Dermatol. 2019, 58(12), 1430. 10.1111/ijd.14455. 30972736

[8] S. C. Peterson , M. Eberl , A. N. Vagnozzi , A. Belkadi , N. A. Veniaminova , M. E. Verhaegen , C. K. Bichakjian , N. L. Ward , A. A. Dlugosz , S. Y. Wong , Cell Stem Cell. 2015, 16(4), 400. 10.1016/j.stem.2015.02.006. 25842978 PMC4387376

[9] G. Y. Wang , J. Wang , M. L. Mancianti , E. H. Epstein , Cancer Cell. 2011, 19(1), 114. 10.1016/j.ccr.2010.11.007. 21215705 PMC3061401

[10] G. Gualdi , P. Monari , P. Calzavara‐Pinton , S. Caravello , F. Fantini , C. Bornacina , F. Specchio , G. Argenziano , V. Simonetti , S. Caccavale , M. la Montagna , R. Cecchi , C. Landi , M. Simonacci , D. Dusi , M. Puviani , A. Zucchi , P. Zampieri , M. A. G. Inchaurraga , F. Savoia , D. Melandri , A. Capo , P. Amerio , Int. J. Dermatol. 2020, 59(3), 377. 10.1111/ijd.14728. 31774173

[11] E. Hudson , H. M. Abu , SAGE Open Med. Case Rep. 2020, 8. 10.1177/2050313X20939481. PMC7370554 32733678

[12] S. Puig , A. Berrocal , Clin. Transl. Oncol. 2015, 17, 497. 10.1007/s12094-014-1272-9. 25643667 PMC4495248

[13] N. Basset‐Seguin , F. Herms , Acta Derm. Venereol. 2020, 100(11), adv00140. 10.2340/00015555-3495. 32346750 PMC9189749

[14] K. Tanese , Curr. Treat. Options Oncol. 2019, 20(2), 13. 10.1007/s11864-019-0610-0. 30741348

[15] A. Angelopoulou , N. Alexandris , E. Konstantinou , K. Mesiakaris , C. Zanidis , K. Farsalinos , K. Poulas , Environ. Res. 2020, 188, 109858. 10.1016/j.envres.2020.109858. 32846644 PMC7309930

[16] N. Kiss , F. Cantoresi , S. Lampitelli , R. Marino , A. Bánvölgyi , N. M. Wikonkál , C. Cantisani , Dermatol. Ther. 2020, 33(6), e14390. 10.1111/dth.14390. 33037759 PMC7646024

[17] M. Chun‐Guang , L. Qi‐Man , Z. Yu‐Yun , C. Li‐Hua , T. Cheng , H. Jian‐De , Ind. J. Dermatol. 2014, 59(6), 575. 10.4103/0019-5154.143520. PMC4248494 25484387

